# Sulfur-Doped Reduced Graphene Oxide for Enhanced Sodium Ion Pseudocapacitance

**DOI:** 10.3390/nano9050752

**Published:** 2019-05-16

**Authors:** Yiting Wang, Mingxiang Hu, Desheng Ai, Hongwei Zhang, Zheng-Hong Huang, Ruitao Lv, Feiyu Kang

**Affiliations:** 1 State Key Laboratory of New Ceramics and Fine Processing, School of Materials Science and Engineering, Tsinghua University, Beijing 100084, China; wangyiti16@mails.tsinghua.edu.cn (Y.W.); frankhu1993@163.com (M.H.); hw-zhang14@mails.tsinghua.edu.cn (H.Z.); zhhuang@tsinghua.edu.cn (Z.-H.H.); fykang@sz.tsinghua.edu.cn (F.K.); 2 Institute of Nuclear and New Energy Technology, Tsinghua University, Beijing 100084, China; 3 Key Laboratory of Advanced Materials (MOE), School of Materials Science and Engineering, Tsinghua University, Beijing 100084, China

**Keywords:** sulfur-doped reduced graphene oxide, sodium-ion pseudocapacitor, cathode materials

## Abstract

Sodium-ion capacitors (NICs) are considered an important candidate for large-scale energy storage in virtue of their superior energy–power properties, as well as availability of rich Na^+^ reserves. To fabricate high-performance NIC electrode material, a hydrothermal method was proposed to synthesize sulfur-doped reduced graphene oxide (SG), which exhibited unique layered structures and showed excellent electrochemical properties with 116 F/g capacitance at 1 A/g as the cathode of NICs from 1.6 V to 4.2 V. At the power–energy density over 5000 W/kg, the SG demonstrated over 100 Wh/kg energy density after 3500 cycles, which indicated its efficient durability and superior power–energy properties. The addition of a sulfur source in the hydrothermal process led to the higher specific surface area and more abundant micropores of SG when compared with those of reduced graphene oxide (rGO), thus SG exhibited much better electrochemical properties than those shown by rGO. Partially substituting surface oxygen-containing groups of rGO with sulfur-containing groups also facilitated the enhanced sodium-ion storage ability of SG by introducing sufficient pseudocapacitance.

## 1. Introduction

Various clean energies (e.g., solar energy, wind energy) have been widely explored to alleviate the environmental impact caused by combustions of fossil fuels. However, the electricity from these resources is usually unstable and needs high-throughput storage. Among different candidates, lithium-ion batteries and capacitors have been considered as the most efficient electrochemical energy storage devices [1,2,3]. However, the consumption of lithium resources has surged upwards with the increasing need for energy storage devices on electric vehicles and portable electronics, which has led to even higher prices and a shortage of the very limited lithium reserves. Grid-scale electrical energy storage applications call for lower-cost battery technology.

Alternatively, the sodium-ion battery (NIB) and sodium-ion capacitor (NIC) have become favorable candidates for the next-generation large-scale energy storage devices in virtue of the similar properties of sodium and lithium, as well as the abundant Na^+^ resources. Tremendous efforts have been made in recent years to search for better electrode materials for NIBs to replace commercial lithium-ion batteries, particularly in the anode side. Among the materials that have attracted the most attention, metal sulfides present higher theoretical specific capacities but suffer with serious volume changes during cycling [4]. Metal oxides present good cycling stability, nevertheless, low specific capacity and poor conductivity limit their practical applications [5,6,7,8]. Two-dimensional (2D) MXenes [9] and metal organic frameworks (MOFs) [10] have also been widely explored in this field, but the rate capacity is still not high enough to meet the increasing demand in practical applications.

Compared to NIB, NIC exhibits a higher capacity rate and longer cycle stability due to the double-layer energy storage system, but the energy density is relatively low [11,12,13,14,15]. In order to achieve a more efficient and high-throughput electrochemical energy storage, developing advanced electrode materials to satisfy the ever-increasing requirement of high energy and power densities is crucial for the NIC systems.

As the most typical 2D material, pristine graphene possesses excellent carrier mobility which has been widely investigated in batteries, supercapacitors, sensors, and catalysts [16,17,18,19,20,21,22,23,24]. As a typical derivative material of graphene, graphene oxide (GO) demonstrates great potential as electrodes for batteries or capacitors because of its low cost and rich functional groups [25,26,27,28].

Two effective means for meeting the demands of advanced carbon-based electrode materials have appeared. One is to optimize the pore structure, including the pore size and the pore distribution [29]; the other involves the introduction of heteroatoms into the carbon network to bring defective sites where the extra faradic redox reactions can occur [30,31].

Heteroatom doping of GO is an efficient way to tailor its structures and functionalities [32,33,34,35]. In particular, it has been confirmed that a pseudocapacitive interaction between the electrolyte ions and the nitrogen-containing functional groups would occur due to nitrogen doping in GO [36,37,38,39,40]. Along with this strategy, and on the basis of some theoretical calculations, some other heteroatoms including oxygen [41], phosphorus [19,42], boron [43,44,45], and silicon [46,47] have been investigated and doped into electrode materials in lithium-ion batteries (LIBs) or other types of electrochemical devices. In addition, Yang et al. [18] directly annealed GO and benzyl disulfide in argon and successfully synthesized sulfur-doped graphene (S-graphene). When being applied in oxygen reduction reactions (ORRs), as-fabricated S-graphene exhibits prominent catalytic activity, long-term stability, and high methanol tolerance in alkaline media, which is attributed to the similar electronegativity of C (2.55) and S (2.58) and the ability of S doping to tailor the atomic charge density and spin density distribution on graphene [18].

As demonstrated in the previous work, introducing S-containing functional groups will create lattice defects in reduced graphene oxide, which provides more active sites where the Faradaic pseudocapacitance occurs. Doping of S gives rise to three types of structural defects in a sulfur-doped graphene network, addressed as pentagon defect (including single (SPent) and double (DPent) pentagon defect), Stone–Wales defect (SW), and tetragon defect (Tetr). The SPent and DPent, together with the Stone–Wales defect are quite stable in an S-doped graphene structure (energy costs of 0.53, 0.64, and 0.55 eV/at, respectively) [48]. These prevailing lattice defects are expected to play an important role in stabilizing curved S-doped pentagon-containing graphene-like planes.

However, the properties of sulfur-doped reduced graphene oxide (SG) as the NIC electrode material and its applications are seldom explored. In this work, we utilized a hydrothermal method to synthesize SG, which exhibited excellent Na^+^ storage ability as the cathode of the NIC. It demonstrated over 100 Wh/kg energy density with a power–energy density over 5000 W/kg after 3500 cycles. The strategy we proposed here may provide some new ideas for designing 2D electrode materials for NICs.

## 2. Materials and Methods

We utilized a modified Hummers’ method to prepare GO, which has been reported in previous work [16]. SG was synthesized via a hydrothermal method. The specific synthesis process of the material was as follows. First, a solution was obtained by adding 20 mg of thioacetamide (TAA) into 80 mL of GO solution (1 mg/mL), and then stirred by magnetic stirring for 30 min. Then, the solution was heated to 180 °C in a 100 mL Teflon-lined stainless steel autoclave and maintained for 12 h. The hydrothermal product was collected by centrifugation, rinsed several times with deionized water and ethanol, and finally freeze-dried. We also synthesized reduced graphene oxide as a comparative sample under the same method (just without the addition of TAA).

The crystalline structures of the as-obtained samples were characterized using X-ray diffraction (XRD, Bruker AXS, Karlsruhe, Germany) with Cu Kα radiation. A field-emission scanning electron microscope (FE-SEM, JEOL JSM-5600, Tokyo, Japan) equipped with an energy dispersive spectrometer (EDS) and a transmission electron microscope (TEM, FEI Tecnai-G2, Hillsboro, OR, USA) were used to observe the morphology of the as-obtained samples. Nitrogen absorption and desorption measurements were conducted on a Belsorp-Max/Mini (MicrotracBEL Corp., Osaka, Japan) system to analyze the pore structures of samples.

To prepare slurries, 90 wt% sample and 10 wt% polyvinylidenediflouride (PVDF) were mixed in N-methylpyrrolidone (NMP) and stirred for 4 h. Then a doctor blade method was used to prepare electrodes by covering the surface of aluminum foils with the slurries. To remove the solvents, electrodes were then dried in a vacuum drying oven at 120 °C for 12 h. The loading mass of the as-obtained electrode was around 0.8 mg/cm^2^. At room temperature, half-cells were assembled in a glove box which was filled with argon. A mixture of 1 M NaClO_4_ in ethylene carbonate (EC) and dimethyl carbonate (DMC) (1:1 vol%) was used as electrolyte. Sodium metal was pressed into foil, which was used as the counter electrode. A land battery test system and an electrochemical workstation (Chenhua, Shanghai, China) were used to perform Galvanostatic charge and discharge (GCD) and Cyclic voltammetry (CV) measurements, respectively. GCD were conducted in the voltage ranging from 1.6 to 4.2 V (vs. Na^+^/Na). CV measurements were conducted with a 0.1 mV s^−1^ scan rate from 1.6 to 4.2 V (vs. Na^+^/Na). To investigate the resistances, electrochemical impedance spectroscopy (EIS) measurements were also performed on the electrochemical workstation with a frequency from 0.01 Hz to 100 kHz. All measurements were performed at room temperature. XRD, Raman, FT-IR, SEM, TEM, CV and GCD tests were followed to check the reproducibility of the proposed system.

## 3. Results and Discussion

### 3.1. Morphology and Structural Characterization

**Figure 1:**
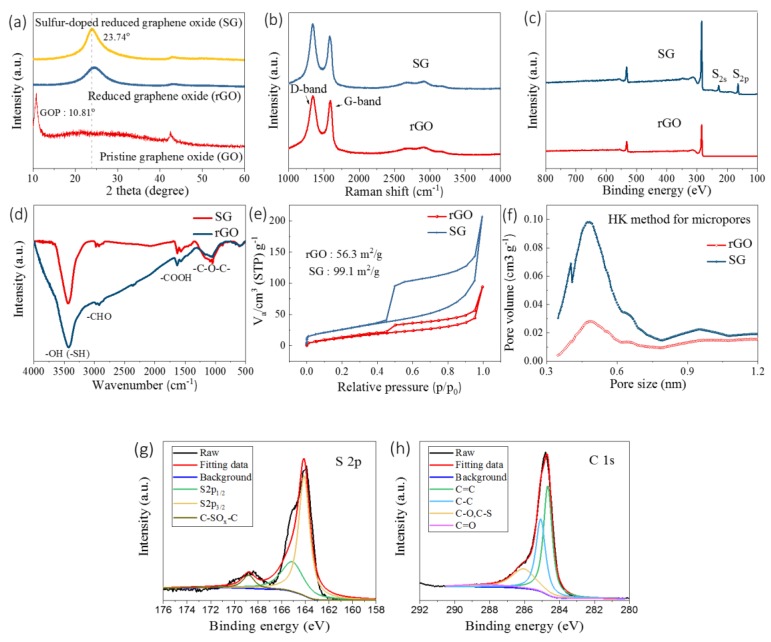
(**a**) X-ray diffraction (XRD) patterns of pristine graphene oxide (GO) and sulfur-doped reduced graphene oxide (SG); (**b**) Raman spectra of reduced graphene oxide (rGO) and SG; (**c**) X-ray photoelectron spectrum (XPS) surveys; (**d**) Fourier-transformed infrared (FT-IR) spectra of rGO and SG; (**e**) N_2_ absorption/desorption isotherms; (**f**) the corresponding pore size distributions of rGO and SG samples; (**g**) High-resolution S 2p X-ray photoelectron spectrum (XPS) spectrum; and (**h**) high-resolution C 1s XPS spectrum of SG.

The phase structures of the as-obtained SG and rGO were characterized using XRD and Raman methods, as demonstrated in Figure 1a,b,c. As the XRD patterns illustrate in Figure 1a, a peak in pristine graphene oxide existed at around 10.81°. It is explained that the excessive oxygen-containing groups on the surface of GO would attribute to a peak in this position [16]. After the hydrothermal treatments with TAA, the as-obtained SG displayed different XRD patterns with two peaks located at 23.74° and 42.78°, which can be indexed to the (002) and (10) diffraction peaks of rGO, respectively [49]. The XRD peaks of SG and rGO showed no significant difference, except for a slight downshift of (002) in the peak position of rGO when compared with that of SG. Furtherly, the defects and graphitization degree of the as-obtained samples were determined using Raman spectra, as demonstrated in Figure 1b. Both rGO and SG possessed two typical broad peaks centered at around 1342 and 1592 cm^−1^, respectively, which are consistent with D-band and G-band in carbon-based materials. The integral intensity ratio of D to G peaks (*I_D_/I_G_*) implies the defect type and defect density of different samples. As illustrated in Figure 1b, the *I_D_/I_G_* ratios rose from 1.09 (rGO) to 1.21 (SG), indicating that SG was more defective because of the doping of the sulfur resource TAA with high-temperature treatment. XPS technique was used to scrutinize the existence of sulfur in SG, as demonstrated in Figure 1c. There only existed the elements C, S, and O in the SG sample, indicating the successful doping of sulfur species [50]. S 2p XPS spectra obviously revealed three distinct peaks corresponding to the S−C bond at 165.09 eV, 164.10 eV, and 168.79 eV, which were identified as the spin−orbit coupling positions of S 2p1/2, S 2p3/2, and C–SOx–C (x = 2,3), respectively, in accordance with previous reports [51,52,53,54]. Meanwhile, high-resolution C1s XPS analysis of SG was deconvoluted into four peaks (Figure 1h), featuring the C=C bond at 284.67 eV, the C–C bond at 285.08 eV, the C–O and/or C–S bond at 286.06 eV, and the C=O bond at 288.37 eV. The bond of C−S (286.06 eV) unraveled in the C 1s XPS spectrum further identified the existence of a C–S bond [31,52].In fact, C–S bonds at the edge or near defects can provide important active sites which benefit the charging of the electrical double-layer and eventually become conducive to the supercapacitive performance [55].

The surface states of rGO and SG were also characterized by the FT-IR absorption spectra, as shown in Figure 1d. Compared to rGO, the peak intensity of SG in the FT-IR spectrum was much stronger, which can be attributed to its additional sulfur-containing functional group. Peaks located at around 1720 cm^−1^ and 1160 cm^−1^ in the FT-IR spectrum of both rGO and SG corresponded to carboxyl (–COOH) and alkoxy (–C–O–C–), respectively. The state of S was further identified according to the featured peaks at 3420 cm^−1^ and 1130 cm^−1^ formed by in-plane bending vibration of S–H bonds and stretching vibration of C–S bonds, respectively [35]. The specific surface areas (SSAs) and related pore distributions of rGO and SG were detected by N_2_ adsorption/desorption technique. As shown in Figure 1e, both curves exhibited type IV hysteresis loops, which indicated that the structure of rGO and SG is highly consistent with materials containing sheet-like structures and slit-shaped pores. With the assistance of density function theory (DFT), it was calculated that the SSAs of rGO and SG were 56.3 m^2^/g and 99.1 m^2^/g, respectively. The relevant pore size distributions are demonstrated in Figure 1f, on the basis of a Horvath-Kawazoe (HK) simulation method. It is obvious that the content of micropores (sized about 0.5 nm) in SG were much higher than those in rGO, leading to the superior electrochemical properties of SG in NICs. The distinct porous structures are related to the different reaction route based on the heteroatom precursors [53]. We utilized thioacetamide (TAA) as a sulfur precursor, thus some pores were generated by the gas released during the hydrothermal process. This possibly accounted for the content difference of micro pores between SG and rGO.

**Figure 2:**
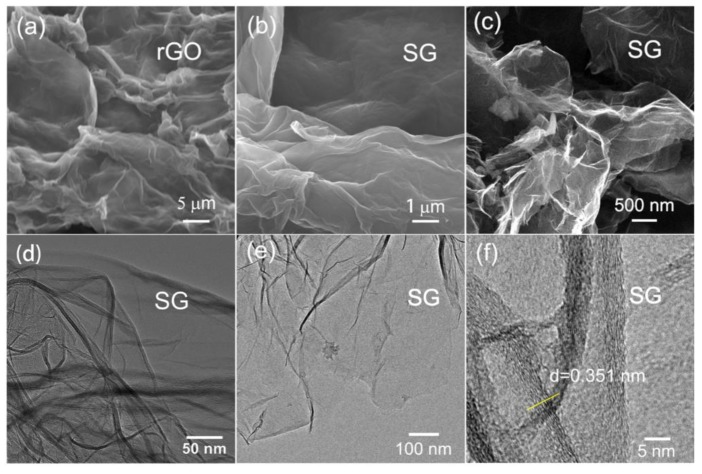
(**a**) Scanning electron microscope (SEM) image of rGO; (**b,c**) SEM images of SG; (**d,e**) Low-resolution transmission electron microscope (TEM) image of SG; (**f**) high-resolution TEM (HRTEM) image and lattice spacing of SG.

The morphologies and microstructures of the as-obtained samples are demonstrated in Figure 2. From Figure 2a, it can be inferred that the rGO sample revealed typical layer-stacked structures. Compared to the structures of rGO, SG exhibited a similar morphology with a partly folded surface, as shown in Figure 2b,c. Transmission electron microscopy (TEM) images shown in Figure 2d,e further proved the layered structures of the as-obtained SG. The high-resolution TEM image in Figure 2f shows the enlarged images of the black margins in Figure 2d,e; it was calculated that the lattice spacing was about 0.351 nm, which is consistent with the XRD analysis.

### 3.2. Electrochemical Properties

**Figure 3:**
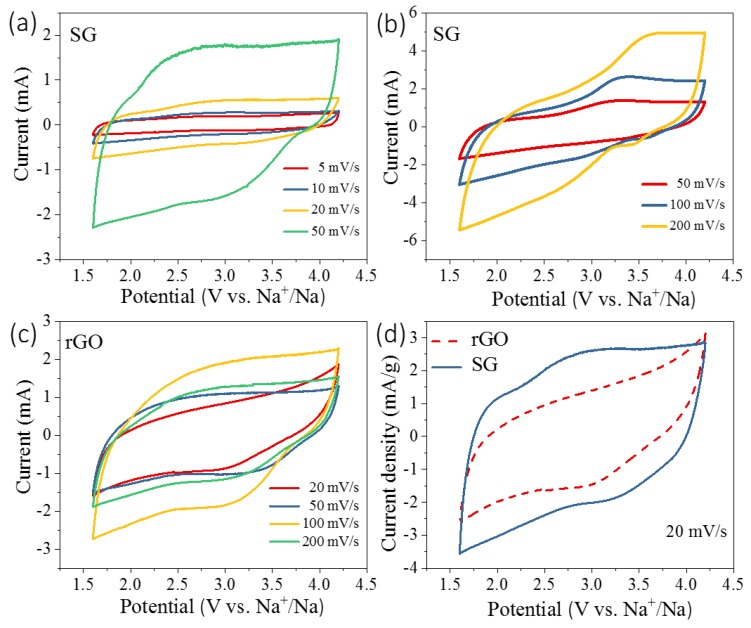
(**a,b**) Cyclic voltammetry (CV) curves of SG at different scan rates; (**c**) CV curves of rGO at different scan rates; and (**d**) comparison of CV curves of SG and rGO samples.

**Figure 4:**
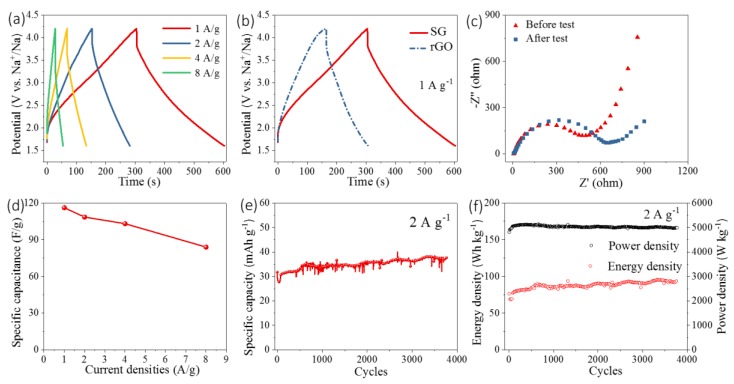
(**a**) Galvanostatic charge and discharge (GCD) curves of SG as the cathode of NICs ranging from 1.6 V to 4.2 V at different current densities; (**b**) comparison of the GCD curves of SG and rGO; (**c**) Nyquist plots of SG before and after electrochemical tests; (**d**) rate capacitances of SG at different current densities; and (**e,f**) Cycle performance of SG at a current density of 2 A/g.

The electrochemical properties of SG and rGO were firstly investigated to elucidate their capacitive behaviors using CV technique, as shown in Figure 3. Figure 3a,b illustrate that the CV curves of SG displayed typical rectangular shapes which are usually observed in supercapacitor-based materials. Besides, there existed two redox peaks at about 2.5 V and 3 V in the anodic and cathodic processes in the CV curves, respectively. These two peaks were caused by the interactions between surface S-containing groups and anions, which is usually defined as pseudocapacitance. Introducing S-containing functional groups creates lattice defects in reduced graphene oxide, which provides more active sites where the Faradaic pseudocapacitance occurs. These S-containing functional groups can also enhance the wettability of the electrode material, which lowers the interface resistance. Compared with rGO, SG becomes more nucleophilic due to the existence of the lone-pair S 2p_z_ electrons. These improved physical properties of SG give rise to better supercapacitive performances. With the increase of scan rates, the areas of CV curves show proportional relationships with their relevant scan rates. Meanwhile, more pronounced polarization would take place when the scan rates increase. Comparing the CV curves of rGO and SG, apart from two pseoducapacitive humps, the absolute area of SG was much higher than that of rGO. As the GCD curves show in Figure 4b, the specific capacitances of SG and rGO were 116 F/g and 58.8 F/g, respectively. With the introduction of S-containing groups, the specific capacitance exhibited a 100% increase. As shown in Figure 4a, SG were tested as the cathode of NICs under 1 A/g, 2 A/g, 4 A/g, and 8 A/g, respectively, at the potential ranging from 1.6 V to 4.2 V.

The capacitance (*C*) can be determined according to the equation below:

$$C=\frac{I\cdot t}{m\cdot U}$$

where *I* is the current, *t* refers to the time, *m* represents the mass of active materials, and *U* is the voltage window. It was calculated that SG could deliver 84.0 F/g, 103.2 F/g, 108.5 F/g, and 116.0 F/g under the current densities of 8 A/g, 4 A/g, 2 A/g, and 1 A/g, respectively (Figure 4d). There was low formation of a solid electrolyte interlayer (SEI) in SG when it worked as the cathode of NICs, which on one hand resulted from the relatively high working potential of NICs, and on the other hand was from the higher standard electrode potential of Na than of Li. However, the EIS analysis in Figure 4c revealed that after the electrochemical properties test, the charge transfer resistance of SG increased to almost 600 Ω from 450 Ω of pristine SG. The increased resistance was mainly caused by the irreversible reactions in the initial cycle. SG possessed a characteristic of good long cyclability and could deliver over 40 mAh/g specific capacity, even after 3500 cycles under 2 A/g. Meanwhile, at the power density of 5000 W/kg, SG also demonstrated over 100 Wh/kg energy density after 3500 cycles. Experiments showed that the capacitances can be well-reproduced.

## 4. Conclusions

In summary, sulfur-doped reduced graphene oxide was synthesized through a simple hydrothermal method. The as-synthesized SG exhibited typical layer-stacked structures with ~100 m^2^/g SSA. As the cathode for NICs, SG delivered almost two times the capacitance of rGO, with 116 F/g capacitance at 1 A/g. Meanwhile, SG demonstrated over 100 Wh/kg energy density at the power–energy density over 5000 W/kg after 3500 cycles. The prominent electrochemical properties of SG can be ascribed to the layered structures, high carrier mobility, and high amount of surface pseudocapacitive functional groups. In virtue of the merits mentioned above, SG and other kinds of doped graphene materials are promising to perform profound functions for the electrochemical energy storage applications.
